# Influence of Asian Dust Particles on Immune Adjuvant Effects and Airway Inflammation in Asthma Model Mice

**DOI:** 10.1371/journal.pone.0111831

**Published:** 2014-11-11

**Authors:** Jun Kurai, Masanari Watanabe, Katsuyuki Tomita, Hiroyuki Sano Akira Yamasaki, Eiji Shimizu

**Affiliations:** 1 Department of Respiratory Medicine and Rheumatology, Tottori University Faculty of Medicine, Yonago, Tottori, Japan; 2 Department of Respiratory Medicine, Yonago Medical Center, Yonago, Tottori, Japan; 3 Department of Respiratory Medicine and Allergology, Kinki University Faculty of Medicine, Osaka-Sayama, Osaka, Japan; The Ohio State University, United States of America

## Abstract

**Objective:**

An Asian dust storm (ADS) contains airborne particles that affect conditions such as asthma, but the mechanism of exacerbation is unclear. The objective of this study was to compare immune adjuvant effects and airway inflammation induced by airborne particles collected on ADS days and the original ADS soil (CJ-1 soil) in asthma model mice.

**Methods:**

Airborne particles were collected on ADS days in western Japan. NC/Nga mice were co-sensitized by intranasal instillation with ADS airborne particles and/or Dermatophagoides farinae (Df), and with CJ-1 soil and/or Df for 5 consecutive days. Df-sensitized mice were stimulated with Df challenge intranasally at 7 days after the last Df sensitization. At 24 hours after challenge, serum allergen specific antibody, differential leukocyte count and inflammatory cytokines in bronchoalveolar lavage fluid (BALF) were measured, and airway inflammation was examined histopathologically.

**Results:**

Co-sensitization with ADS airborne particles and Df increased the neutrophil and eosinophil counts in BALF. Augmentation of airway inflammation was also observed in peribronchiolar and perivascular lung areas. Df-specific serum IgE was significantly elevated by ADS airborne particles, but not by CJ-1 soil. Levels of interleukin (IL)-5, IL-13, IL-6, and macrophage inflammatory protein-2 were higher in BALF in mice treated with ADS airborne particles.

**Conclusion:**

These results suggest that substances attached to ADS airborne particles that are not in the original ADS soil may play important roles in immune adjuvant effects and airway inflammation.

## Introduction

Many studies have shown that exposure to particulate matter is associated with respiratory and cardiovascular morbidity or mortality [1] [2]. Asian dust storms (ADSs) originating from deserts in Mongolia, Northern China, and Kazakhstan often disperse dust over East Asia from spring until late autumn [3] [4] and ADS airborne particles increase the atmospheric particulate matter. The original ADS soil is transported over a long distance and becomes attached to chemical species such as sulfate (SO_4_^2−^) and nitrate (NO^3−^), and to microbial agents [5], [6]. Therefore, ADS airborne particles have a wide variety of substances on their surface.

We have shown that ADS exposure can aggravate upper and lower tract respiratory symptoms and pulmonary dysfunction in adult patients with asthma [7] [8] [9]. Other studies have also found an association of an ADS with an increased risk of hospitalization in children with asthma [10] [11] [12]. These studies suggest that an ADS can exacerbate asthma, but the underlying mechanism remains unclear.

ADS airborne particles increase airway inflammation and have immune adjuvant effects in ovalbumin (OVA)-induced asthma model mice [13] [14] [15]. However, these studies used Asian sand dust after heat treatment at 360°C for 30 min to inactivate certain substances (microbiological material) attached on particles. Our previous study showed that ADS airborne particles induced production of IL-8 in THP-G8 cells, but this effect did not occur with the original ADS soil. Thus, substances attached to ADS airborne particles may provoke exacerbation of asthma. However, the OVA-induced asthma mouse model uses aluminum as an adjuvant, but aluminum is an important constituent of Asian sand dust [13]. The innovative asthma mouse model developed by Shibamori et al. shows allergic asthma-like reactions after intranasal sensitization by Dermatophagoides farinae (Df) [16]. Thus, these mice may be more useful than the OVA-induced model because the Df-induced asthma model does not require an adjuvant such as aluminum.

In this study, we explored the differences in immune adjuvant effects and airway inflammation between ADS airborne particles and original ADS soil, with the goal of investigating the mechanism of exacerbation of asthma caused by an ADS. The study was performed in Df-induced asthma model mice and without heating of the particles to avoid degradation of attached substances.

## Materials and Methods

### Animals

Specific pathogen-free 7-week-old male NC/Nga mice were purchased from Japan SLC Inc. (Hamamatsu, Japan) and acclimatized for 7 days before the start of the study. Animals were kept in a storage room at a constant temperature of 22°C and illumination with 12-hour light/dark cycles. Animals were fed standard animal chow daily and had access to drinking water ad libitum. The experimental protocols were approved by the Institutional Animal Care and Use Committee, Faculty of Medicine, Tottori University (protocol number 12-Y-55).

### Preparation of ADS airborne particles and original ADS soil

CJ-1 soil from the China Loess Plateau, the original ADS soil in the Tengger Desert and Huining (Gansu Province), was obtained from the National Research Center for Environmental Analysis and Measurement (Ibaraki, Japan) in 2002. Airborne particles were collected in Tottori on ADS days from March 8 to March 11 in 2013 using a high-volume air sampler (HV-1000R; Shibata Co., Tokyo, Japan) which was fixed on the rooftop of a building. ADS airborne particles were separated according to their aerodynamic diameters into 5 filters (<1.1, 1.1–2.0, 2.0–3.3, 3.3–7.0, >7.0 µm) and each filter was dried in a desiccator before and after sampling to be weighted. In this study, we used ADS airborne particles whose size was 3.3–7.0 µm. CJ-1 soil and ADS airborne particles were sterilized at 121°C for 30 min in an autoclave (Tomy SX-300; Tomy Co., Tokyo, Japan) and stored in a freezer at −20 C° to prevent growth of bacteria and fungi. For administration to mice, CJ-1 soil and ADS airborne particles were diluted with normal saline (NS).

### Experimental protocol

NC/Nga mice were sensitized to Df (Greer Laboratories Inc., Lenoir, NC, USA) as described elsewhere [16]. After a 7-day acclimatization period, mice were divided randomly into six groups (n = 8 per group). For sensitization, mice anesthetized by isoflurane inhalation were intranasally instilled with Df crude extract (50 µg) diluted with 25 µl of NS for 5 consecutive days (days 0–4). Df-sensitized mice were challenged intranasally with Df at 7 days after the last Df sensitization (day 11) and sacrificed 24 h after the Df challenge. In the control group, NS was administered instead of Df sensitization.

**Figure 1:**
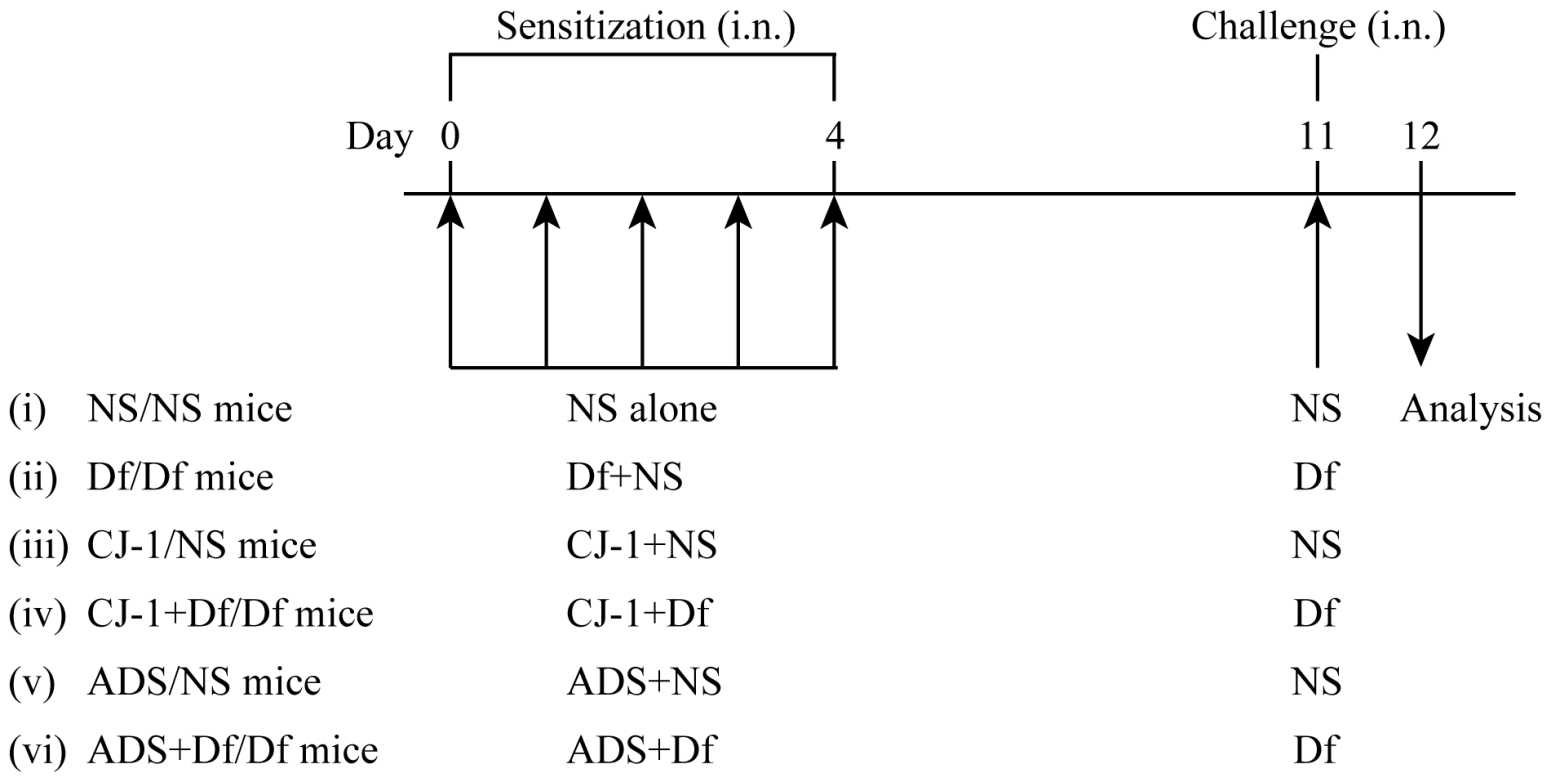
Experimental protocol. NC/Nga mice were given intranasal (i.n.) applications of a mixture (ADS airborne particles and/or Df, or CJ-1 soil and/or Df) as sensitization for 5 consecutive days (days 0–4). At 7 days after the last allergen sensitization, mice were challenged by allergen, followed by collection of BALF, lung tissue, and serum. Mice were divided into 6 groups: NS/NS, Df/Df, CJ-1/NS, CJ-1+Df/Df, ADS/NS, and ADS+Df/Df.

To observe immune adjuvant effects and airway inflammation induced by CJ-1 soil or ADS airborne particles (0.1 mg/25 µl of NS respectively), mice were co-sensitized by intranasal instillation of ADS airborne particles and/or Df, or CJ-1 soil and/or Df for 5 consecutive days (days 0–4) (Figure 1). The six groups were (i) NS/NS mice: sensitized with NS and challenged by NS; (ii) Df/Df mice: sensitized with Df and challenged by Df; (iii) CJ-1/NS mice: sensitized with CJ-1 soil and challenged by NS; (iv) CJ-1+Df/Df mice: co-sensitized with Df and CJ-1 soil and challenged by Df; (v) ADS/NS mice: sensitized with ADS airborne particles and challenged by NS; and (vi) ADS+Df/Df mice: co-sensitized with Df and ADS airborne particles and challenged by Df.

### BALF procedure

After the mice were anesthetized with isoflurane, tracheas were cannulated. BALF was subsequently obtained with instillation of 1.0 ml NS into the lungs, along with gentle handling to maximize BALF recovery. BALF from each mouse was centrifuged at 300×g for 5 min at 4°C. The cell pellet was used for cell count and the supernatant was used for cytokine analysis. Total cells diluted in Turk's fluid were counted using a hemocytometer. The differential leukocyte count was obtained by microscopic evaluation and quantitative analysis of methanol-fixed cytospin preparations stained with Diff Quick (Fisher Scientific, Pittsburgh, PA, USA).

### Histological examination

Mice were euthanized by injection of pentobarbital. Lungs were inflation-fixed at 25 cm of water pressure with 10% formalin for 5 min and immersed in the same fixative. Tissues were fixed for 24 h at 4°C and processed using standard methods for paraffin-embedded blocks. Fixed lung tissues were embedded in paraffin and each section was stained with hematoxylin-eosin (H&E) and periodic acid-Schiff/Alcian blue (PAS-AB).

### Enzyme-linked immunosorbent assay for serum total and Df-specific immunoglobulin

Total IgE and total IgG2a levels were measured with OptEIA Mouse kits (BD Pharmingen, San Diego, CA, USA). Df-specific IgE and Df-specific IgG2a levels were detected as previously described [17]. Briefly, 96-well plates were coated with a 50 µg/ml solution of Df and incubated overnight at 4°C. The content of each well was removed and the plate was washed with wash buffer (BD Pharmingen). Serum dilutions were 1/4 and 1/3000 for measuring Df-specific IgE and Df-specific IgG2a, respectively. The diluted serum was added to each well and incubated for 2 h at room temperature. Streptavidin-horseradish peroxidase conjugate (BD Pharmingen) was added to each well and incubated for 30 min at room temperature. The plate was developed with tetramethylbenzidine (100 µl/well) in the dark at room temperature for 30 min. The optical density (450 nm) was read with Sunrise, a microplate calibrated reader (Tecan Group, Japan), running the program XFluor4 (Tecan Group).

### Cytokine and chemokine levels in BALF

Interferon (IFN)-γ, interleukin (IL)-13, IL-5, IL-6, keratinocyte-derived chemokine (KC/CXCL1), macrophage inflammatory protein (MIP)-2 (KC/CXCL1 and MIP-2/CXCL2 are murine homologues of human IL-8), thymus and activation-regulated chemokine (TARC/CCL17), and interferon gamma-induced protein (IP)-10/CXCL10 levels were measured by sandwich ELISA (R&D Systems Europe, Abingdon, UK). BALF dilutions were 1/5 for IL-13, IL-5, IL-6, KC/CXCL1, MIP-2/CXCL2, TARC/CCL17, and IP-10/CXCL10. BALF was undiluted for IFN-γ.

### Statistical analysis

Data are expressed as mean and standard deviation (SD). Comparisons between groups were made by one-way ANOVA. Calculations were performed with GraphPadPrism ver. 5.02 (GraphPad Software, San Diego, CA, USA). P<0.05 was considered to be significant.

## Results

### Co-sensitization with ADS airborne particles and Df augments airway inflammation

**Figure 2:**
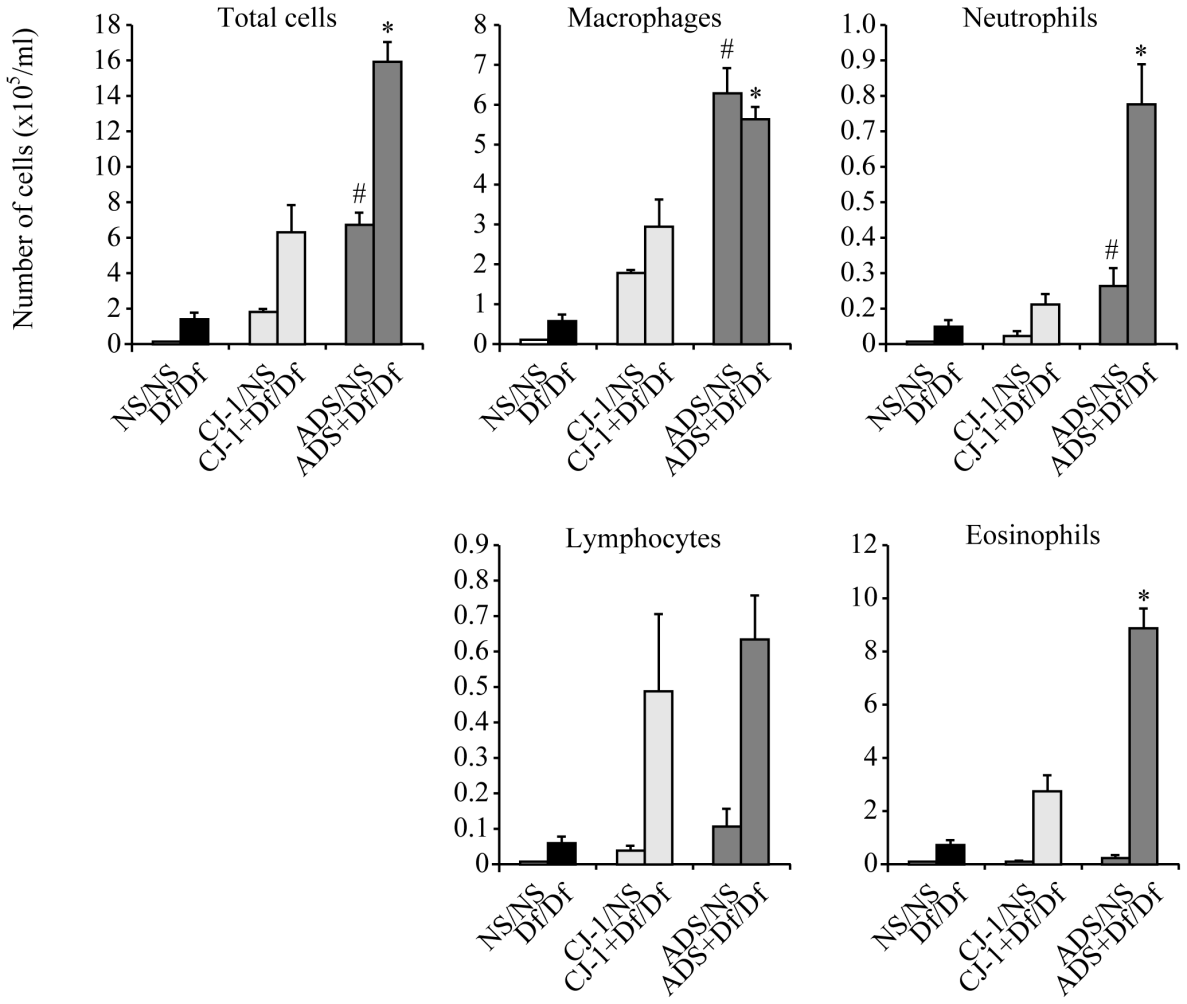
Total and differential leukocyte counts in BALF. The cell count in BALF was obtained from 24 h after allergen challenge on day 11. The differential leukocyte count was identified as eosinophils, macrophages, neutrophils, and lymphocytes. Total cell counts in ADS+Df/Df mice were increased significantly compared to CJ-1+Df/Df mice. Data are expressed as mean ±SD of 8 mice per group. *p<0.05 vs. CJ-1+Df/Df group, # p<0.05 vs. CJ-1/NS group.

Df/Df mice had a significant increase in total cell count in BALF compared to control NS/NS mice (p<0.05). In mice co-sensitized with CJ-1 soil and Df (CJ-1+Df/Df mice), the total cell count in BALF showed a significant 4.6-fold increase compared to Df/Df mice due to elevation of macrophages, lymphocytes and eosinophils (Figure 2). Mice co-sensitized with ADS airborne particles and Df (ADS+Df/Df mice) had greater enhancement of airway inflammation compared to CJ-1+Df/Df mice, and the total cell count was significantly increased by 2.5-fold in ADS+Df/Df mice (Figure 2). The increased cell count was consistent across macrophages, eosinophils and neutrophils, with significant elevations of 1.9-, 3.3-, and 6.2-fold, respectively, in ADS+Df/Df mice compared to CJ-1+Df/Df mice (Figure 2). The increase of neutrophils was most noticeable in the differential leukocyte count. There was no significant increase in lymphocytes.

### Effects of ADS airborne particles on histopathological changes in lung

**Figure 3:**
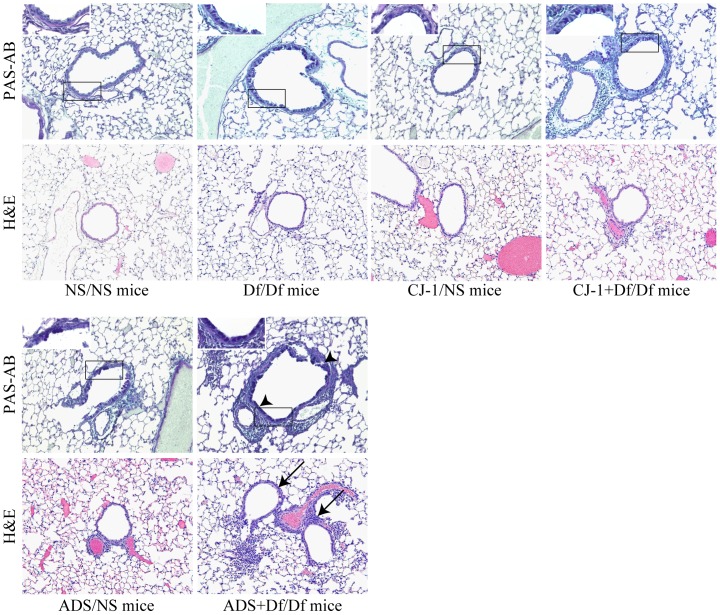
Effects of CJ-1 soil and ADS airborne particles on histopathological changes in lung. Light photomicrographs of representative lung sections stained with hematoxylin-eosin (magnification ×200) and periodic acid-Schiff/Alcian blue (magnification ×200). Representative light photomicrographs of NS/NS mice, Df/Df mice, CJ-1/NS mice, CJ-1+Df/Df mice, ADS/NS mice and ADS+Df/Df mice. Arrows show peribronchiolar and perivascular mixed inflammatory cell infiltration in ADS+Df/Df mice. Inset figure is higher magnification of the boxed area (magnification ×400). Arrow heads indicate the accumulation of mucus in the airway epithelial cells in ADS+Df/Df mice.

To determine the histopathological effects of co-sensitization with ADS airborne particles and Df on the airway, lung specimens were evaluated by staining with H&E and PAS-AB. CJ-1+Df/Df and ADS+Df/Df mice had greater peribronchiolar and perivascular inflammatory cell infiltration than CJ-1/NS and NS/AD mice, respectively. Greater airway inflammation was also apparent in ADS+Df/Df mice compared with CJ-1+Df/Df mice (Figure 3, H&E staining). Df/Df mice showed relatively weaker inflammatory responses than ADS+Df/Df and CJ-1+Df/Df mice. These histopathological findings were consistent with the BALF analysis. Furthermore, there was a markedly greater mucus cell metaplasia with increased amounts of PAS-AB-stained mucosubstances on the surface epithelium in ADS+Df/Df mice compared with CJ-1+Df/Df mice (Figure 3, PAS-AB staining).

### Co-sensitization with ADS airborne particles and Df potentiates immune adjuvant effects

**Figure 4:**
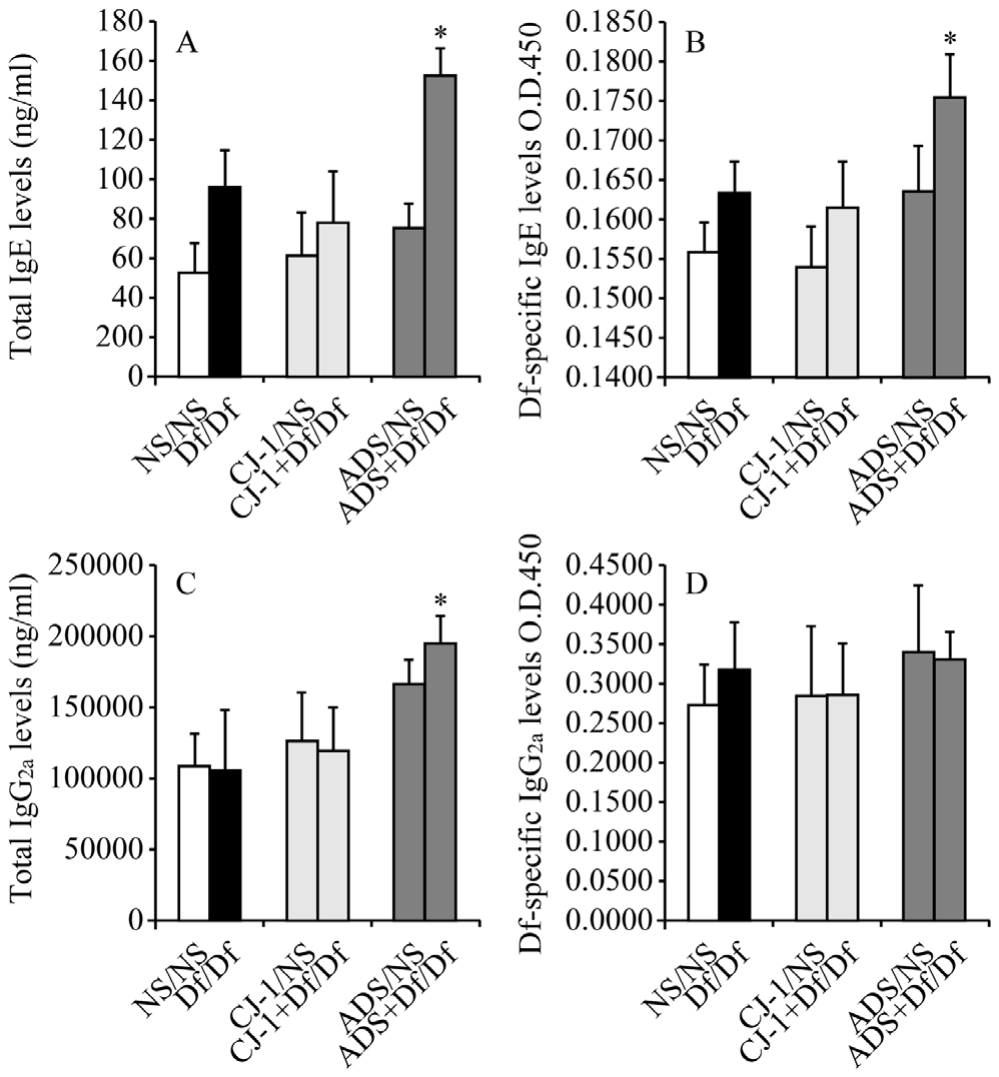
Total IgE, total IgG2a, Df-specific IgE, and Df-specific IgG2a levels in serum. Serum total IgE (A) and total IgG2a (C) were measured by ELISA and are shown as serum concentrations. Df-specific IgE (B) and Df-specific IgG2a (D) were measured based on optical density. Results are expressed as mean ±SD of 6 mice per group. *p<0.05 vs. CJ-1+Df/Df group.

To examine the immune adjuvant effects of CJ-1 soil and ADS airborne particles in Df-sensitized mice, serum immunoglobulin levels were evaluated on day 12. ADS+Df/Df mice had significantly increased total IgE and total IgG2a (1.9- and 1.6-fold, respectively, as immunoglobulin concentrations, p<0.05) and Df-specific IgE (p<0.05) compared to CJ-1+Df/Df mice (Figure 4). In contrast, co-sensitization with CJ-1 soil and Df (CJ-1+Df/Df mice) had no effect on total IgE, total IgG2a, Df-specific IgE, and Df-specific IgG2a levels (Figure 4). These data indicate that ADS airborne particles recruit inflammatory cells into lung tissues and potentiate immune adjuvant effects, whereas CJ-1 soil does not have these effects.

### Co-sensitization with ADS airborne particles and Df induces production of Th2 and inflammatory cytokines

**Figure 5:**
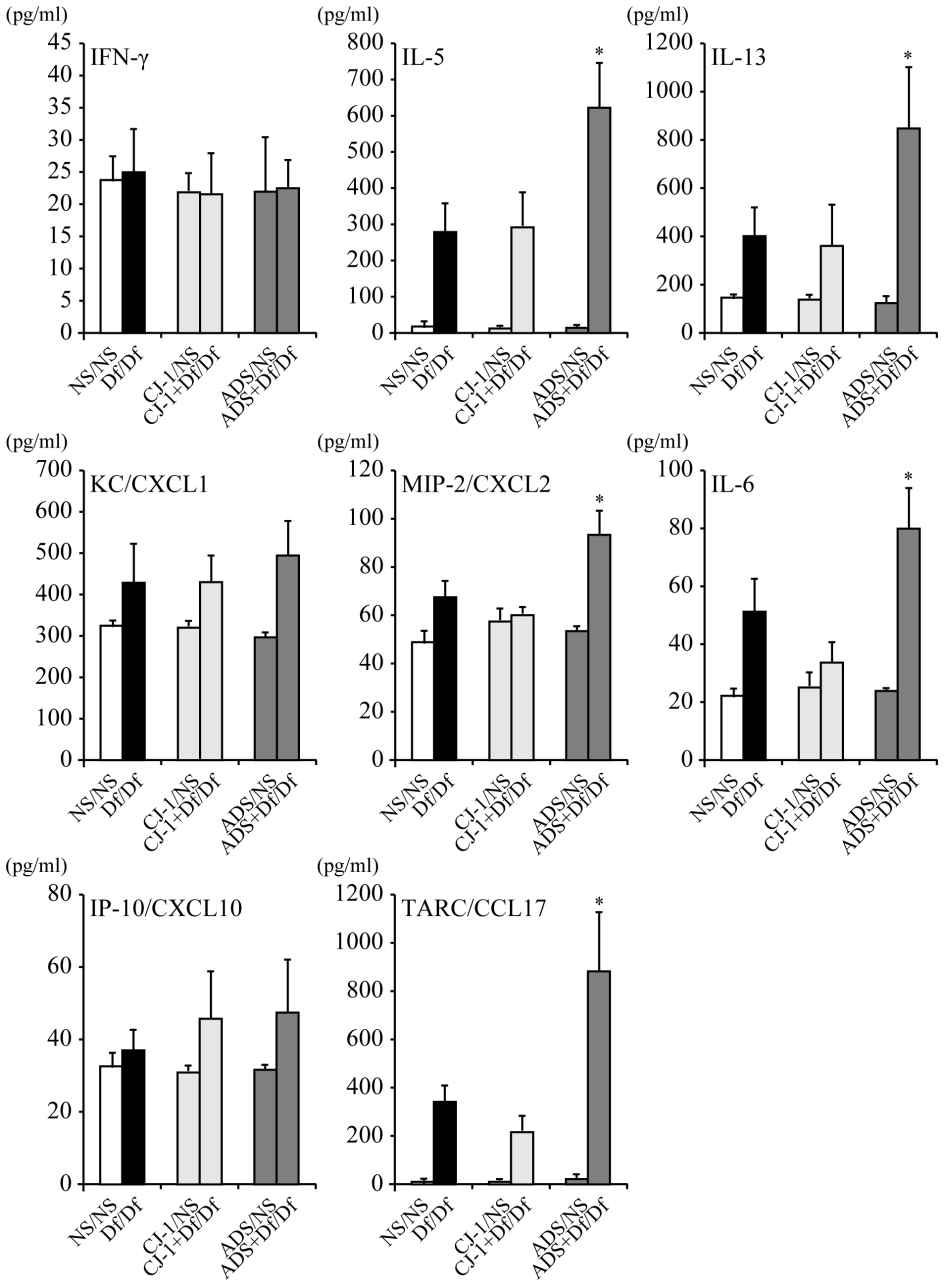
Cytokines and chemokines levels in BALF. BALF cytokine and chemokine expression profiles were analyzed by ELISA for IFN-γ, IL-5, IL-13, IP-10/CXCL10, TARC/CCL17, KC/CXCL1, MIP-2/CXCL2 and IL-6. Data for each group are expressed as mean ±SD of 6 mice per group. *p<0.05 vs. CJ-1+Df/Df groups.

To investigate the mechanisms through which ADS airborne particles cause an allergic airway response in Df-induced asthma model mice, cytokine levels in BALF were measured. In parallel with the inflammatory cell recruitment in BALF, ADS airborne particles induced production of several important cytokines for asthma. BALF from ADS+Df/Df mice had higher levels of Th2 cytokines (IL-5, IL-13); inflammatory cytokines (IL-6, MIP-2/CXCL2), except for KC/CXCL1, a neutrophil activation chemokine; and a Th2 chemokine (TARC/CCL17), compared to CJ-1+Df/Df and Df/Df mice (Figure 5). In contrast to ADS+Df/Df mice, CJ-1+Df/Df mice did not have significant increases in cytokine and chemokine levels compared to control Df/Df mice. A Th1 cytokine (IFN-γ) and a Th1 chemokine (IP-10/CXCL10) showed no increase after administration of CJ-1 soil or ADS airborne particles.

## Discussion

This study shows that airborne particles collected on ADS days have significantly different effects as an immune adjuvant and on airway inflammation compared to original ADS soil. The ADS airborne particles significantly increased the number of neutrophils and eosinophils in BALF, and significantly augmented airway inflammation with mucus hypersecretion compared to the original ADS soil. The particles also induced significant elevation of serum total IgE, total IgG2a, and Df-specific IgE, but the original ADS soil did not do so. ADS airborne particles also increased production of inflammatory cytokines (IL-6 and MIP-2/CXCL2) and Th2 cytokines (IL-5 and IL-13). These results suggest that substances attached to ADS airborne particles augment airway inflammation and increase immune adjuvant effects.

Air pollutants augment human airway inflammation through effects on IL-8 and IL-6 [18] [19] [9], and IL-8 and IL-6 have important roles in neutrophilic airway inflammation in patients with asthma [20] [21] [22]. There were significant increases in MIP-2/CXCL2 (a murine homologue of IL-8) and IL-6 and in the neutrophil count in BALF of ADS+Df/Df mice compared to CJ-1+Df/Df mice. This implies that ADS airborne particles have a stronger effect than CJ-1 soil in inducing neutrophilic airway inflammation via MIP-2/CXCL2 and IL-6 elevation. Honda et al. showed that IL-8 and IL-6 increase in a dose-dependent manner after exposure of airway epithelial cells to ADS airborne particles *in vitro* [23], and also found that these particles did not induce IL-8 and IL-6 after cauterization to eliminate attached substances.

These results suggest that the different effects of ADS airborne particles and original ADS soil may depend on substances such as chemicals and microorganisms attached to the particles. It is unclear which of these substances has most toxicity in human health. Inhaled LPS is associated with neutrophilic airway inflammation in healthy subjects and patients with asthma [24] [25]; and He et al. found that ADS airborne particles have LPS on their surface [26], but that the original ADS soil does not contain LPS. Thus, LPS on the particles may have an important role in inducing neutrophilic airway inflammation via MIP-2/CXCL2 and IL-6 elevation.

Co-sensitization with ADS airborne particles and Df increased the number of eosinophils and elevated IL-5 and IL-13 in BALF. The increase in neutrophils in BALF was also greater than that of eosinophils in ADS+Df/Df mice. Thus, exacerbation of asthma by an ADS may be more closely linked to neutrophilic airway inflammation. ADS airborne particles also increased serum total IgE, total IgG2a and Df-specific IgE, indicating that these particles have immune adjuvant effects. In contrast, CJ-1 soil did not increase Th2 cytokines (IL-5, IL-13) or immunoglobulins, which suggests that substances on ADS airborne particles contribute to production of Th2 cytokines, total IgE, total IgG2a and Df-specific IgE.

OVA is often used as an allergen in asthma model mice, but OVA is not the major allergen in asthma. OVA-induced model mice are also sensitized with intraperitoneal injection of aluminum as an adjuvant, but aluminum is an important component of Asian sand dust [13]. This suggests that it may be difficult to assess the adverse effect of ADS airborne particles in OVA-induced model mice. Previous study also showed that even an amorphous silica, which is a major component of Asian sand dust, may also potentiate the immune adjuvant effect by itself [27], though we could not detect it in our study. We cannot exclude an immune adjuvant effect of aluminum in ADS airborne particles, but administration of these particles was performed by intranasal instillation, rather than intraperitoneal injection (as used in OVA-induced model mice). Thus, we believe that Df-induced NC/Nga asthma model mice are useful for accurate evaluation of the influence of ADS airborne particles because this mouse model does not require an adjuvant and is challenged with an allergen that closely resembles that in human asthma.

There are several limitations in this study. First, we did not analyze the composition of the ADS airborne particles in detail. Thus, we cannot identify the attached substances that increased immune adjuvant effects and airway inflammation. Second, we were unable to collect sufficient amounts of ADS airborne particles for use in the challenge, and this prevented investigation of the effects of the particles in this phase. Third, we did not evaluate airway hyperresponsiveness (AHR), though previous study shows significant increases in AHR after intranasal administration of Df in this asthma mice model [16]. Therefore, the present study could not show that airborne particles collected on ADS days may provoke exacerbation of asthma functionally. Finally, although use of NC/Nga mice strains combined with Df sensitization and challenge protocols is likely to be meaningful, human patients with asthma are a heterogeneous population defined by unique interactions between genetic and environmental factors. Hence, exposure to ADS airborne particles may lead to different types of health damage in different individuals.

In summary, this is the first report to show that airborne particles collected on ADS days in western Japan, but not the original ADS soil, have immune adjuvant effects, augment airway inflammation via an increase of neutrophils and promote mucus hypersecretion. These results suggest that substances attached to ADS airborne particles play important roles in exacerbating asthma upon exposure to an ADS. Further studies are required to investigate the mechanisms how substances attached to ADS airborne particles augment airway inflammation, and which substances play more important role.
