# Correction: Influence of Asian Dust Particles on Immune Adjuvant Effects and Airway Inflammation in Asthma Model Mice

**DOI:** 10.1371/journal.pone.0114879

**Published:** 2014-11-26

**Authors:** The PLOS ONE Staff

There are multiple errors in the author byline. Please see the correct author byline here.

Jun Kurai^1^, Masanari Watanabe^1^, Katsuyuki Tomita^2^, Hiroyuki Sano^3^, Akira Yamasaki^1^, Eiji Shimizu^1^

1 Department of Respiratory Medicine and Rheumatology, Tottori University Faculty of Medicine, Yonago, Tottori, Japan,

2 Department of Respiratory Medicine, Yonago Medical Center, Yonago, Tottori, Japan,

3 Department of Respiratory Medicine and Allergology, Kinki University Faculty of Medicine, Osaka-Sayama, Osaka, Japan

The correct citation is: Kurai J, Watanabe M, Tomita K, Hiroyuki S, Yamasaki A, et al. (2014) Influence of Asian Dust Particles on Immune Adjuvant Effects and Airway Inflammation in Asthma Model Mice. PLoS ONE 9(11): e111831. doi:10.1371/journal.pone.0111831
